# Regulation of Spatiotemporal Patterns by Biological Variability: General Principles and Applications to *Dictyostelium discoideum*

**DOI:** 10.1371/journal.pcbi.1004367

**Published:** 2015-11-12

**Authors:** Miriam Grace, Marc-Thorsten Hütt

**Affiliations:** School of Engineering and Science, Jacobs University Bremen, Bremen, Germany; Rice University, UNITED STATES

## Abstract

Spatiotemporal patterns often emerge from local interactions in a self-organizing fashion. In biology, the resulting patterns are also subject to the influence of the systematic differences between the system’s constituents (biological variability). This regulation of spatiotemporal patterns by biological variability is the topic of our review. We discuss several examples of correlations between cell properties and the self-organized spatiotemporal patterns, together with their relevance for biology. Our guiding, illustrative example will be spiral waves of cAMP in a colony of *Dictyostelium discoideum* cells. Analogous processes take place in diverse situations (such as cardiac tissue, where spiral waves occur in potentially fatal ventricular fibrillation) so a deeper understanding of this additional layer of self-organized pattern formation would be beneficial to a wide range of applications. One of the most striking differences between pattern-forming systems in physics or chemistry and those in biology is the potential importance of variability. In the former, system components are essentially identical with random fluctuations determining the details of the self-organization process and the resulting patterns. In biology, due to variability, the properties of potentially very few cells can have a driving influence on the resulting asymptotic collective state of the colony. Variability is one means of implementing a few-element control on the collective mode. Regulatory architectures, parameters of signaling cascades, and properties of structure formation processes can be "reverse-engineered" from observed spatiotemporal patterns, as different types of regulation and forms of interactions between the constituents can lead to markedly different correlations. The power of this biology-inspired view of pattern formation lies in building a bridge between two scales: the patterns as a collective state of a very large number of cells on the one hand, and the internal parameters of the single cells on the other.

## Author Summary

Pattern formation is abundant in nature—from the rich ornaments of sea shells and the diversity of animal coat patterns to the myriad of fractal structures in biology and pattern-forming colonies of bacteria. Particularly fascinating are patterns changing with time, *spatiotemporal* patterns, like propagating waves and aggregation streams. Bacteria form large branched and nested aggregation-like patterns to immobilize themselves against water flow. The individual amoeba in *Dictyostelium discoideum* colonies initiates a transition to a collective multicellular state via a quorum-sensing form of communication—a cAMP signal propagating through the community in the form of spiral waves—and the subsequent chemotactic response of the cells leads to branch-like aggregation streams. The theoretical principle underlying most of these spatial and spatiotemporal patterns is *self-organization*, in which local interactions lead to patterns as large-scale collective”modes” of the system. Over more than half a century, these patterns have been classified and analyzed according to a”physics paradigm,” investigating such questions as how parameters regulate the transitions among patterns, which (types of) interactions lead to such large-scale patterns, and whether there are "critical parameter values" marking the sharp, spontaneous onset of patterns. A fundamental discovery has been that simple local interaction rules can lead to complex large-scale patterns. The specific pattern "layouts" (i.e., their spatial arrangement and their geometric constraints) have received less attention. However, there is a major difference between patterns in physics and chemistry on the one hand and patterns in biology on the other: in biology, patterns often have an important functional role for the biological system and can be considered to be under evolutionary selection. From this perspective, we can expect that individual biological elements exert some control on the emerging patterns. Here we explore spiral wave patterns as a prominent example to illustrate the regulation of spatiotemporal patterns by biological variability. We propose a new approach to studying spatiotemporal data in biology: analyzing the correlation between the spatial distribution of the constituents’ properties and the features of the spatiotemporal pattern. This general concept is illustrated by simulated patterns and experimental data of a model organism of biological pattern formation, the slime mold *Dictyostelium discoideum*. We introduce patterns starting from Turing (stripe and spot) patterns, together with target waves and spiral waves. The biological relevance of these patterns is illustrated by snapshots from real and theoretical biological systems. The principles of spiral wave formation are first explored in a stylized cellular automaton model and then reproduced in a model of *Dictyostelium* signaling. The shaping of spatiotemporal patterns by biological variability (i.e., by a spatial distribution of cell-to-cell differences) is demonstrated, focusing on two *Dictyostelium* models. Building up on this foundation, we then discuss in more detail how the nonlinearities in biological models translate the distribution of cell properties into pattern events, leaving characteristic geometric signatures.

## Introduction

### Purpose of this review

**Fig 1:**
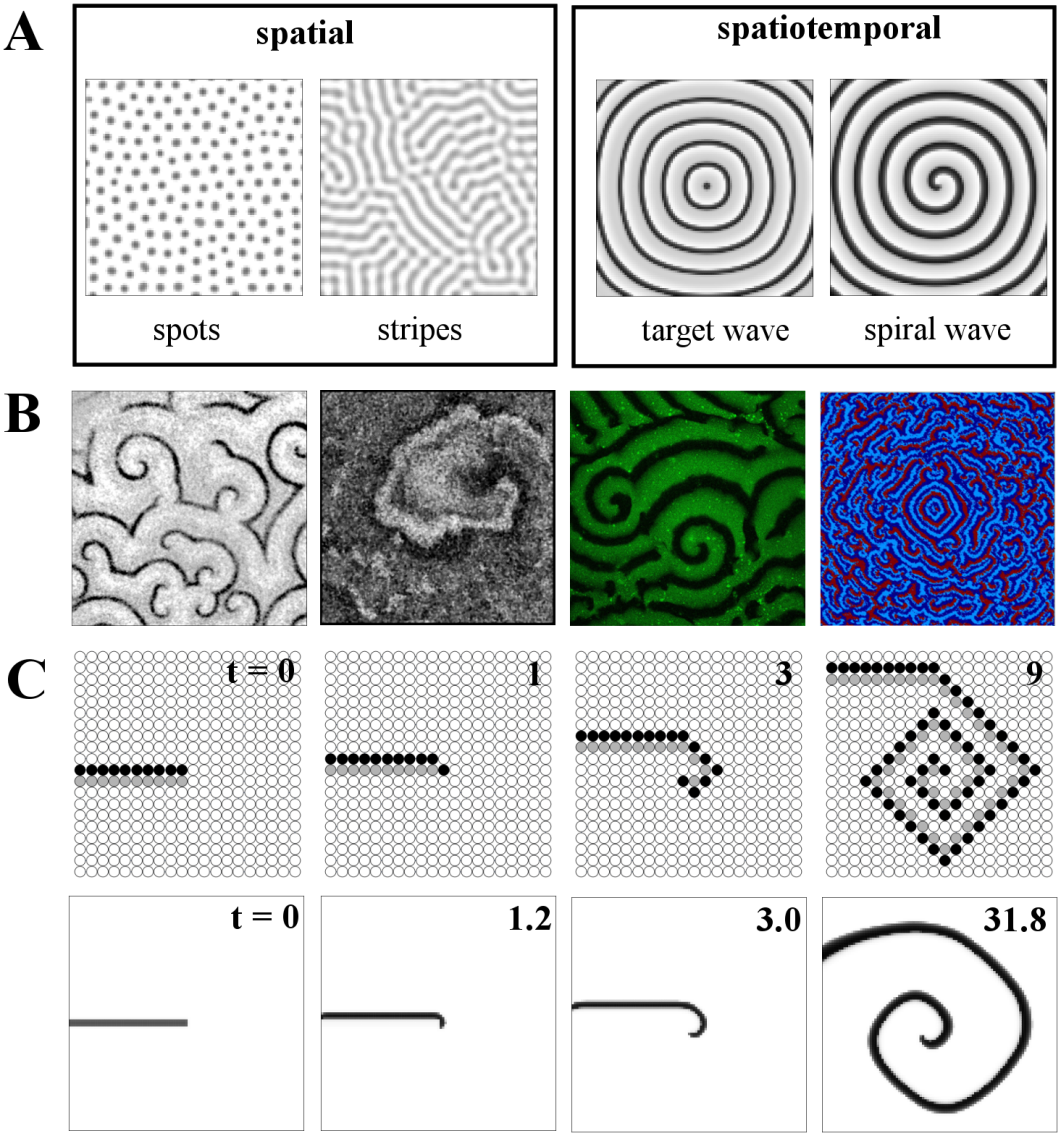
Introduction to pattern types and spiral wave formation. A: (Left to right) spatial and spatiotemporal pattern examples. Spot and stripe Turing patterns both in coupled Schnakenberg elements; target wave formation from a central pacemaker and established spiral wave, both in coupled FitzHugh-Nagumo oscillators. B: Snapshots of spiral wave patterns from diverse biological systems: (left to right) cAMP signaling in a *Dictyostelium discoideum* colony, local contraction in neonatal rat cardiac monolayer cultures, MinD protein density in a lipid bilayer and simulated cytokine levels in a two-dimensional grid of cells. See Acknowledgments for image sources. C (upper row): The update rules of the minimal three-state cellular automaton model lead to spiral wave formation, when applied to an open wave front (consisting of a layer of excited cells, depicted in black, and an adjacent layer of refractory cells, depicted in gray). Lower row: a similar numerical experiment for the model from [6,7].

Patterns in nature have attracted attention for centuries because of their complexity and regularity. A great leap forward in the theoretical understanding of their development from simple interacting constituents was made by Alan Turing in his classic work on morphogenesis [1]. The static "Turing" patterns that develop may take the forms of, among others, spots or stripes, famously reminiscent of those found on animal coats. A simple reaction-diffusion model that can display Turing patterns is the Schnakenberg model (Fig 1A) [2,3]. An analogous mechanism has recently been identified in the form of a cellular interaction network in zebrafish, responsible for its skin patterns [4], and another involved in the mammalian palate [5].

Turing patterns represent one of the major categories of patterns that may be observed. Another important and widespread type is the travelling wave; such patterns are periodic and vary dynamically with time. These wave patterns manifest in biology in situations such as the circular "target" wavefronts produced by the sinoatrial node in the heart, or the open-ended, spiral waves of signaling found in the development of the frog *Xenopus laevis* [8]. Examples of both these wave types are shown in Fig 1A. While target waves require an oscillating "pacemaker" cell in their center, spiral waves have a self-sustaining spiral core and typically originate from the breaking of the wavefront of target waves. The deep link between excitability and wave propagation allows us to look at the underlying mechanisms in an algorithmic fashion: A highly stylized, minimal model of excitable dynamics can reproduce both the schematics of wave propagation and the basic principle of the transition from an open wave front to a self-sustained spiral (see Fig 1C). In this review, we are particularly concerned with understanding aspects of such transitions: in particular, how they are regulated by features of the nonlinear relationships governing the system at hand.

Spatiotemporal patterns have been a highly successful perspective in the study of complex systems for two main reasons: (1) Patterns often fall into a small number of generic categories (e.g., aggregation patterns or propagating waves [9]). They provide fundamental links between local interaction rules and large-scale collective behaviors [10]. The types of interactions and the nonlinearities of the reactions establishing such large-scale patterns are ubiquitous, with reaction-diffusion systems providing the most prominent example; see, e.g., [11]. The prediction derived from the observation of patterns of biochemical agents and corresponding biochemical mechanisms implementing a reaction-diffusion system (e.g., in hydra development and head-region regeneration [12]) constitutes another example of the enormous power that lies in the observation of patterns in real systems and the subsequent dissection (or deconstruction) of these patterns towards an identification of local interaction rules guided by our understanding of self-organization. (2) Patterns are abstract, generic objects that can establish (or make visible) unexpected parallels between diverse biological, technological, and social systems. The ubiquity of spiral wave patterns across many systems and many scales, from molecular-level interactions to ecosystem-wide responses [13], is a good example of this unifying capacity of a pattern perspective. Fig 1B contains some examples of spiral waves in real biological systems.

As high-throughput analysis techniques provide ever more quantitative and often spatially resolved biological data (see, e.g., [14–16] as examples of such technological advances), we now have the means to explore the merits of a biology-inspired view, in which the (statistical) details of the patterns matter. The essential features of a biologically oriented perspective on pattern formation are (firstly) that in the majority of pattern-forming biological systems, the patterns often, though not exclusively (see, e.g., [17] for a critique of an indiscriminately adaptionist perspective on biology), serve a purpose and therefore can be considered to be evolutionarily optimized. A variety of examples exist: the size of spiral waves regulates the size of multicellular aggregates in subsequent stages of aggregation in the slime mold *Dictyostelium discoideum*. Bacteria stabilize themselves via aggregation against water flow. Another slime mold, *Physarum plasmodium*, employs pattern formation to sample space for food sources. Even circadian rhythms in plants can have a complex underlying spatiotemporal organization, leading to propagating waves along leaves, coordinating photosynthetic activity [18]. The stomatal cells of plants respond with complex patterns (termed "stomatal patchiness") to changes in the environment, which effectively implement a density classification algorithm [19].

Noise has always been at the forefront of interest in the analysis of nonlinear systems. For biological systems, over the last few years, intrinsic and extrinsic noise in gene expression [20–22] and in cell fate decisions [23,24] has been investigated. While the variability we refer to here represents fixed differences in cell-to-cell properties, varying, if at all, on a much larger time scale than the resulting patterns, the "extrinsic noise" in these works contains both variability and the collective noise (dynamically) acting upon all cells simultaneously. This distinction is important, as the distribution of cell properties can thus be tuned to achieve certain preferential collective states. Furthermore, some interesting theoretical work has emerged from studying the interplay of noise and variability on pattern formation (see "Background"). Similar to these influences of noise, variability can also shape patterns. As these differences between the constituents are "hard-wired" into the system, we expect a tight evolutionary control on the shaping of patterns by variability.

Secondly, it is plausible, and supported by diverse evidence [25–29], that in biology, the exact spatial layout of the patterns (among the diverse pattern arrangements possible in this systemic configuration) is selected by the distribution of cell properties (i.e., by biological variability), rather than solely—like in physics or chemistry—by spontaneous fluctuations [30]. In this way, spatiotemporal patterns are becoming ever more relevant for computational biology and systems biology because the intracellular "implementation" of such optimized collective states can be investigated. The patterns can serve as a "microscope" for the underlying single-cell mechanisms of regulation.

Understanding how variability (i.e., the magnitude of cell-to-cell differences) shapes collective states (i.e., the emerging patterns) is instrumental to a biology-oriented view of self-organization. We here review the role of variability in the self-organization of biological systems. Our key example is the establishment of spiral wave patterns in excitable media, which is used to model such phenomena as, e.g., the self-organization of *Dictyostelium discoideum* [15,31–33], cardiac arrhythmia [34] and even the epidemic spreading of infectious diseases (see, e.g., [35,36]). We thus explore the relationship between variability in cell properties and features of spiral wave patterns for a variety of mathematical models.

### Basic principles of spiral wave formation and the influence of biological variability

In biology, variability in the form of cell-to-cell differences (in some biologically relevant parameter) can be expected to contribute more significantly than stochastic noise to the heterogeneity in a biological system (see, e.g., [29] for experimental evidence and [37–39] for theoretical arguments). The potential importance of variability in biological pattern formation is one of the most striking differences between biological systems and those in physics or chemistry, where system components are essentially identical and random fluctuations are the only factor determining the details of the self-organization process and the resulting patterns. This has far-reaching implications for our understanding of biological systems, in which patterns are linked to function. The patterns frequently constitute a (precursor of a) collective systemic mode and are thus subject to evolutionary selection. Variability may be seen as a mechanism by which individual elements shape these collective modes.

The authors in [33] have convincingly made this case for *Dictyostelium*, relating the strength of a regulatory feedback loop to the spatial density of spiral-wave patterns. By studying mutants in key components of this loop, the authors systematically varied this intrinsic parameter and observed the corresponding changes in the patterns. Their main finding is that wild-type feedback strength is optimized to yield maximal aggregation territory size. This allows for optimally sized basins of attraction for the consecutive aggregation process of the *Dictyostelium* cells in the formation of the multicellular stage capable of spore generation, which completes the developmental cycle. It is also mechanistically plausible that variability in cell properties could be an important driving mechanism behind spatiotemporal patterns in biological systems, as the degree of such differences determines the capacity of the cells to locally synchronize and, consequently, form patterns on a larger spatial scale. Variability, the existing pattern of cell-to-cell differences, is constant in time. It gives rise to heterogeneity in pattern-forming substances (e.g., in the distribution of a chemotactic substance). This heterogeneity, in turn, leads to the establishment of self-organized patterns. In *Dictyostelium*, the concept of a genetically encoded "developmental path," a starvation-induced transition of the individual cells through different dynamical regimes of behavior, initially the excitable rest state, then oscillatory, before once more becoming excitable, provides a framework through which the effects of heterogeneity can be implemented in a realistic theoretical setup (see [40] and "Models").

We start out with an example to illustrate how the distribution of cell properties can influence self-organized spatiotemporal patterns. Spiral waves can be generated in a variety of different ways: one of the most accessible basic mechanisms can be shown with an extremely simple model of excitable dynamics. Fig 1 introduces the main mechanistic idea of spiral wave pattern formation, illustrates their ubiquity in biological systems and provides a first example of how they are influenced by biological variability. The model used is a three-state cellular automaton on a two-dimensional lattice. Each lattice site is either susceptible *S*, excited *E*, or refractory *R*. An excitable (susceptible) element *S* acquires the state *E*, if there is an excitation *E* in its four-element neighborhood; a refractory element *R* returns to the susceptible state *S* after one time step (see "Models" for details). In Fig 1C, upper row, an open-ended wave front (first snapshot) curls up to form a spiral wave. In spite of the geometrical artifacts due to the simple four-element neighborhood and the simplistic state space, the general principle is clearly visible: the initial conditions of a wavefront segment (black elements) directly followed by a layer of refractory lattice sites (gray elements) trigger two forms of patterns, a propagating wave front (oriented upwards in the figure) and the open end of the wave front giving rise to first an additional excited element and then to a curved wave segment, which subsequently propagates outwards in a spiral shape.

A more sophisticated model (by [7]), which we will refer to as the "Levine model," brings the whole scenario much closer to biological reality. It was originally formulated to account for the basic mechanisms behind spiral wave formation in *Dictyostelium discoideum*. Technically, it is an ordinary differential equation (ODE)—cellular automaton hybrid. It uses effective degrees of freedom (excitability, refractory phase), along with biologically plausible variables (the local concentration of the intercellular signaling substance, cAMP) and parameters (e.g., the feedback strength, describing how an external cAMP signal affects cAMP production) to account for the most important properties of the actual biological system. This model has also been used in [37] and [33]. The lower row in Fig 1C shows a similar open-wavefront simulation using the Levine hybrid model, indicating that even with this more complex model, the behavior of spiral wave formation is qualitatively the same.

As these two examples demonstrate, spirals, and more generally any pattern in excitable media, may arise through different mechanisms. In general, a propagating circular wavefront must be interrupted. This may occur when such a wavefront encounters an obstacle in the form of refractory or less excitable elements (S1 Fig), when a target wave is generated inside another wave and can only partially propagate, or through special initial conditions which mimic this process, as in our first examples. Though their origins are still not always fully understood, spiral waves are ubiquitous in biological systems. Fig 1B gives examples of spatial snapshots from diverse biological situations. The first is cAMP concentrations, spreading through a colony of *Dictyostelium* cells prior to the aggregation phase of the amoeba’s life cycle (image from the database described in [44]; see also [33]). The second snapshot shows a spiral wave of electric activity forming in cardiac tissue: here, in neonatal rat cell cultures, imaged using a dye-free modality that tracks local contraction (see [41]). Such spiral waves are pathological, and their breakup appears to lead to cardiac arrhythmia. The third image shows spiral waves of protein density at a sub-cellular scale: the proteins MinD and MinE, which have a key function in *Escherichia coli* cell division, are here reconstituted in vitro on a supported lipid bilayer in the presence of ATP as the energy source (see [42]). The last snapshot in Fig 1B shows a simulation of spiral waves occurring in the context of signaling in mammalian tissue. The cytokine production of cells distributed on a regular 2-D lattice is shown. Suitable amounts of noise change the wave structure to the point where spiral waves appear in the tissue (see [43]).

In *Dictyostelium*, such spiral waves represent a chemotactic signal guiding each cell towards an aggregation site, at which the formation of a multicellular structure is initiated. The distribution of spiral waves thus directly affects the fate of each cell. The spiral wave density (qualitatively speaking) translates into the cell counts of the multicellular mounds. With spiral waves, a pathological dynamical state of cardiac tissue, identifying the spatial distributions of cell properties that lead to reduced spiral wave probability would be advantageous.

The growing accessibility of *Dictyostelium* mutant data and their resulting pattern-level phenotypes, through studies such as [44], also enhance the prospects of gaining insight into pattern formation in this model organism via mathematical models that incorporate detailed and realistic biological knowledge. Table 1 contains some examples of biological properties in *Dictyostelium*, with their corresponding methods of implementation in a computer model and the potential effects on patterns that result.

**Table 1:** Sources of variability affecting details of macroscale patterns in *Dictyostelium* and their representations in computer-based mathematical models.

Biological quantity	Implementation in a computer model	Potential effect on patterns	Reference
cell density	general fluctuations	initialization of patterns	[6]
cell cycle phase	desynchronization on a "developmental path"	transition from target to spiral waves	[25,40]
excitability	spontaneous firing	change of spiral wave density with feedback strength	[33]
cell motility	agent-based simulations	cell sorting	[45]
response to chemotactic signal	variability on coupling strength	target-spiral competition	[46–48]

These quantities are, of course, interdependent. Cell density [6] and excitability [33], for example, vary with the current stage in the cell cycle [25,40]. That responses of *Dictyostelium* to chemotactic stimuli are cell-specific has been shown in [29]. One method of incorporating variability into *Dictyostelium* models is the use of agent-based models, with applications to processes such as cell sorting [45]. The role of coupling strength was investigated in [46] and [47]; effects such as target-spiral competition were shown in [48].

Different cell properties will influence the exact layout of the emerging patterns in different ways. Similarly, different forms of cellular interactions and signal processing will modulate the effects of this biological variability in different ways. When such interdependences are well understood, the statistical properties of the patterns can thus serve as a "microscope" for the underlying principles of regulation.

## Effect of Variability: A Model Study

The impact of variability on spiral wave patterns is best visualized by an event perspective, in which target wave centers and spiral wave tips are considered "spatiotemporal pattern events." The temporal sequences and spatial distributions of these pattern events can then be compared with the spatial distribution of cell properties. A cluster of oscillatory elements is a likely candidate for emission of target waves. The additional pattern events emerging from this region are then a consequence of the proximity to other such pacemaker regions and the "roughness" of the propagating wave front, which is the result of the sequences of excitability encountered by the propagating wave front—up to the point at which target waves break up into spirals, in one possible mechanism.

As an illustration of the wide range of geometrical influences biological variability has on the emerging layout of spiral wave patterns, we use different models of excitable media (with varying levels of biological detail), simulate spiral wave patterns, reconstruct events (using the methods from [49]), and thus translate the spatiotemporal patterns into a "pattern event plot," in which such underlying geometries are clearly discernible.

How do these "event plots" work? Using algorithms acting upon the space-time cube of the (simulated or measured) data, the centers of target waves, as well as the centers and orientations of spiral waves are identified (details about the algorithm are provided in [49]). Such "event plot" representations of spatiotemporal patterns as sequences of events are not new and can be employed even in the case of homogeneous systems. In [50], they have been employed to study spiral breakup in calcium dynamics. In [33], the identification of spiral wave tips via phase singularities was a prerequisite for studying changes of pattern statistics with the strength of the cAMP feedback loop. However, here, in the presence of variability, the geometric constraints on pattern events arising from the specific nonlinearities become visible. In [25], this has been worked out in detail for the receptor desensitization-based ODE model of *Dictyostelium* signaling, referred to here as the "Goldbeter model" [40].

We performed such pattern-based investigations for the "hodgepodge machine" [51,52], the FitzHugh-Nagumo oscillator as a generic model of excitable dynamics (S2 Fig) [39,53,54] and the *Dictyostelium* models from [7] and [40]. We discuss the results from the latter two models in detail here. The underlying cell property is, on a general level, the level of excitability of the individual elements forming the coupled system. The distribution of this property and its dynamical features, possibly modulated by other properties, determine the development of spiral waves.

The hodgepodge machine from [51] is a more gradual version of the three-state "forest fire" cellular automaton. Its *n* states (a typical value of *n* is 100) are traversed in a monotonously increasing fashion. Two parameters, the "excitability" and the "sensitivity," dictate the impact of neighboring cells (sensitivity) and the steepness of the excitatory response (excitability), respectively. This minimalistic cellular automaton model of excitable dynamics displays high positive correlations between target waves and the distribution of excitability, as well as between spiral waves and the distribution of sensitivity [52].

**Fig 2:**
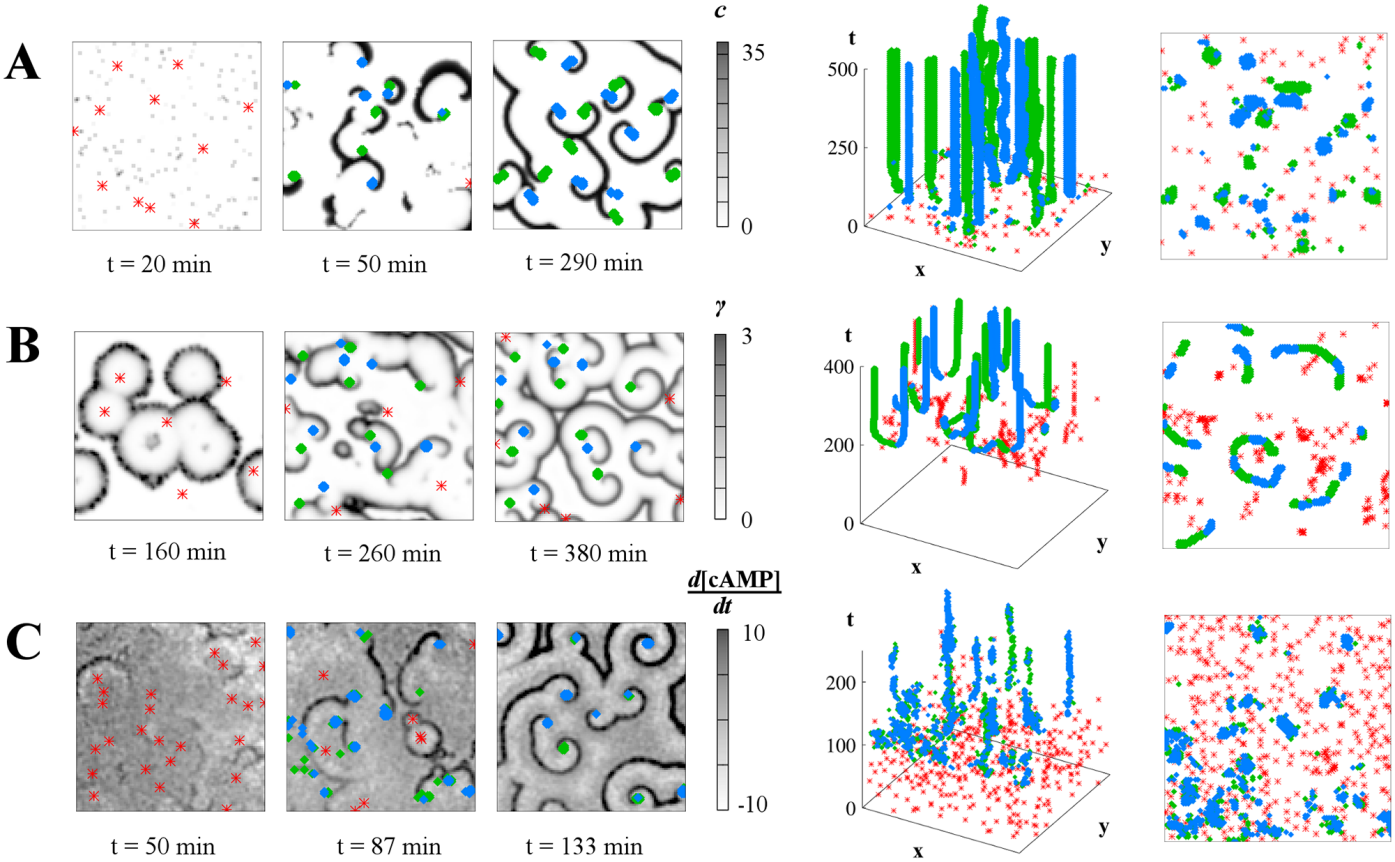
Event identification in simulated and real spiral wave patterns. Left column: snapshots of the lattices; middle column: corresponding space-time event plots; right column: top-down views of the event plots. Target wave origins are red asterisks, left- and right-handed spiral waves are blue and green diamonds, respectively. A: In the "Levine" model, spiral waves evolve early on due to colliding wavefronts, without a sustained target-wave phase. B: Development of spiral waves from the interaction of target waves in the "Goldbeter" model with a developmental path. C: Development of spiral waves from target waves in experimental *Dictyostelium discoideum* data. All simulated lattices are 100x100 and experimental data is rescaled to the same size.

In [39] we showed that a systematic regulation of spatiotemporal patterns by biological variability can be found even in more minimal models than the *Dictyostelium* models discussed here. As a simple and abstracted model of an excitable medium, we studied a lattice of diffusively coupled FitzHugh-Nagumo (FHN) oscillators embedded into a "developmental-path" framework. In this minimal model of spiral wave generation, we could explore the predictability of spatiotemporal patterns from cell properties as a function of desynchronization (or "spread") of cells along the developmental path and the drift speed of cell properties on the path. These two parameters interact to result in systematically different routes towards fully established patterns, as well as strikingly different correlations between cell properties and pattern features [39]. The routes to spiral formation thus depend upon the magnitude and form of variability entering the system. These findings can be seen as providing an event-oriented overview of pattern formation, arising from a symbolic encoding of two basic underlying pattern elements, target waves and spiral waves, that direct this process. The simulation results shown in S2A Fig have been obtained with a "static" version of a FHN lattice (i.e., a lattice of diffusively coupled FHN oscillators without a developmental path). Spirals arise early on from the breaking of target waves at regions of lower excitability, as demonstrated in a similar, minimal setup in S1 Fig. In contrast, S2B Fig contains one exemplary pattern of the diverse routes towards spiral waves which can be obtained in the developmental path version of the FHN lattice. This variant displays a similar sequence of events to the developmental-path Goldbeter *Dictyostelium* model and real *Dictyostelium* data in Fig 2B and 2C, respectively. A high density of spiral tips results, apparently through the "wave-in-wave" mechanism. In both setups, the final pattern is likely determined by the interacting effects of pattern elements in these complex event landscapes (see [48]). These contrasting pattern event sequences illustrate the great versatility of this simple model.

**Fig 3:**
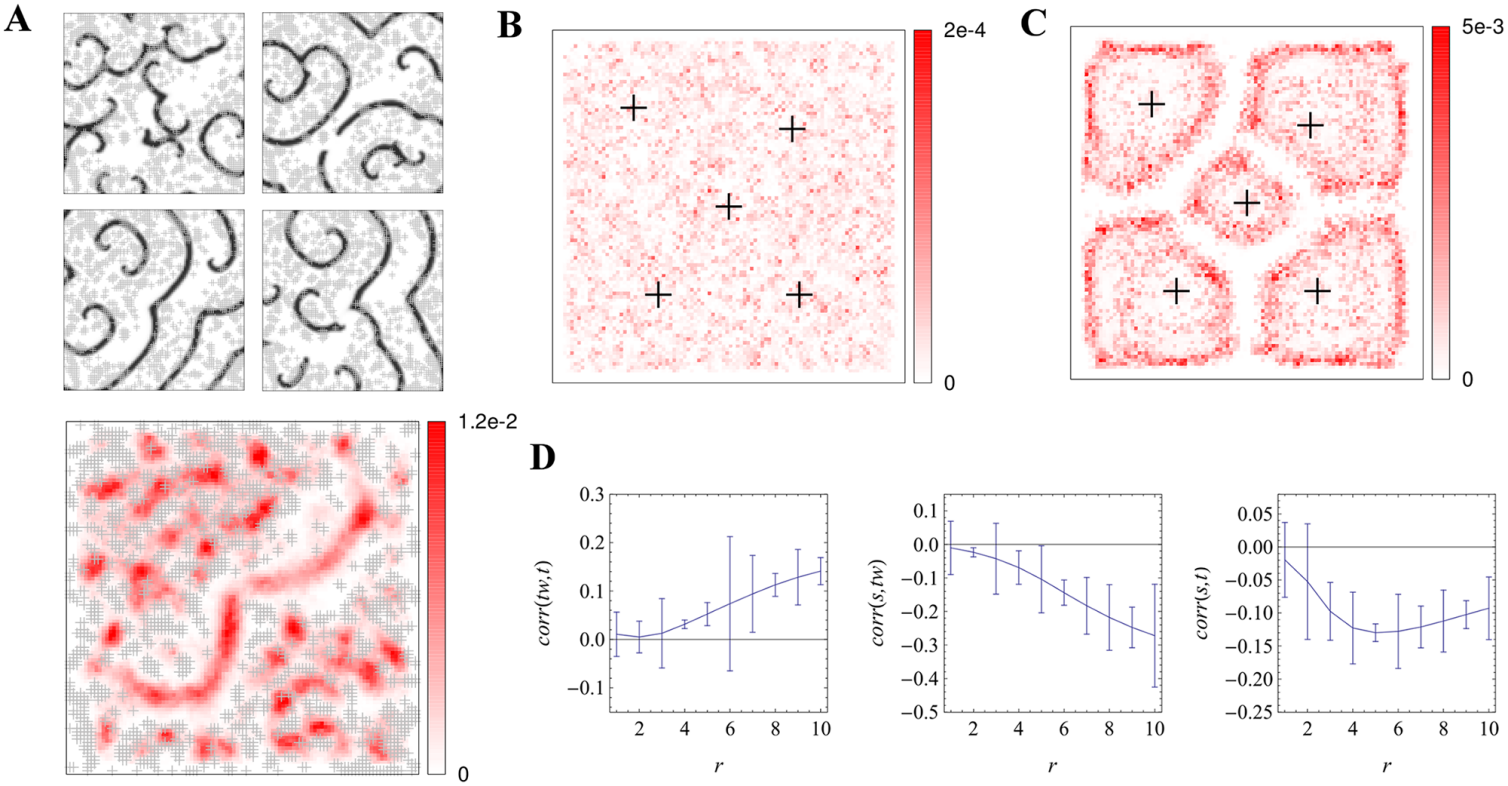
Effect of "pacemaker" location in the "Levine" model from [6,7]. Pacemaker locations are shown as grey or black crosses and spiral tip occupancy in the red scale. A: A conserved pacemaker pattern leads to different spiral wave patterns of excitability (upper row). Spiral tip occupancy over 1,000 runs favors locations free of pacemakers. The probability of observing a spiral wave at a particular spatial site is determined by the spatial distribution of cell properties. A simple pacemaker grouping gives rise to a less coherent, more variable tip occupancy in the Levine model compared to the clear geometric shaping in the "Goldbeter" model (C), based on 250 runs. D: Quantification of the relationships between pattern types in the Goldbeter model: over 100 runs, the correlation coefficient of (left to right) target wave origins to time offsets on the developmental path, spiral tips to target origins and spiral tips to time offsets; against the radius of the Gaussian filter.

Figs 2 and 3 illustrate the main features of these results. In Fig 2 (see also S2 Fig) the general method of pattern event reconstruction and pattern event plots is demonstrated, both for simulated (A and B) and real (C) *Dictyostelium* data. The underlying geometry of the pattern events, as well as similarities and differences between the spatiotemporal arrangements of events, become clearly discernible in these event plots. The effects of the two different models for *Dictyostelium* pattern formation are visible here: the striking geometrical shaping of the arrangement of spiral wave in the Goldbeter model from [40] (2B) contrasts with the conversion of a target wave pattern into a dense spiral wave pattern for the Levine model from [7] (2A). In the latter, the cell property we vary is the capacity to spontaneously enter the excited state and the mechanism of spiral development appears to be based on the breaking of a target wavefront by an unexcitable region. The geometric details of the possible mechanism are explored in [37]. In the experimental *Dictyostelium* data in Fig 2C, spirals form at a later stage, likely due to the interacting target wavefronts that precede their appearance. This event sequence closely resembles that in Fig 2B.

The two *Dictyostelium* models display very different geometric relationships between pattern events. The pairwise appearance of (left- and right-handed) spirals is more pronounced in Fig 2A than in Fig 2B. Both models, as well as the experimental data (Fig 2C), show a clear target wave phase preceding the spiral formation, although this phase is most pronounced in the Goldbeter model in 2B. Also, there are strong differences in spiral wave density (the number of spiral waves per area). However, even at first glance, the biologically more detailed model (Fig 2B) shows a much stronger geometric arrangement of spiral waves than the more schematic model (Fig 2A).

In our previous work on the "Levine model" from [7], we observed a pronounced anti-correlation between spiral waves and a key cell property, the firing rate, and particularly a clustering of spiral wave tips in regions devoid of spontaneously firing "pacemaker" cells. Similar results are shown here in Fig 3A: the spatial locations of the pacemakers remain fixed (grey crosses) and we study the distribution of spiral wave tips across a large number of simulation runs (red-scale signal plotted in the lower row). Although it is hard to discern any clear relationship between pacemaker locations and spiral tip occupancy for single runs (upper row), averaging occupancy over many runs shows the greater density in the central "avenue" free of pacemakers: a clearly visible anticorrelation between the spatial distribution of pacemaker cells and the spatial distribution of spiral wave probabilities. Some features of this distribution can also be understood from a simple geometrical model based on triplets of pacemaker cells [37].

The clearest geometrical shaping of the spiral waves is observed in the Goldbeter model from [40], where variability is coupled to the dynamics via a developmental path. The developmental path moves each element through different dynamical regimes: (non-excitable) *steady state* → *excitable* → *oscillatory* → *excitable*. There, spiral wave tips cluster at characteristic distances from their parent target wave centers: the tips map out the Voronoi diagram around a specific fraction of cells functioning as effective pacemaker cells. These effective pacemakers are those cells that are in the oscillatory regime when the majority of other cells are already in the excitable regime, thus ensuring wave propagation [25]. The pattern events accompanying the evolution of a sample Goldbeter lattice are highlighted in the accompanying animation (S1 Video).

In the top-down (i.e., time-collapsed) views of the event plots, also shown in Fig 2, these geometrical constraints among the pattern elements become apparent. In particular, in the Goldbeter model, the Voronoi diagram-like relationship between the spiral tips and their parent target wave origins are suggested even on this single-run level. Comparing simulation results for the three models shown in Figs 2, 3 and S2 Fig, it is striking how different these geometrical constraints in the emerging spatiotemporal patterns are, both in the characteristic sequence of events and in the spatial density and distribution of asymptotic patterns. This simple example already shows the potential capacity of spatiotemporal patterns to discriminate between possible underlying mechanisms of regulation. However, it should be noted that these examples are based on specific parameter constellations, and a complete evaluation of the model-specific differences requires a comprehensive exploration of the parameter spaces.

In Fig 3B and 3C, we continue our comparison of the Levine and Goldbeter models by exploring their behavior in response to the same initial pacemaker pattern, our cell property here. With the same fixed distribution of five pacemakers, we allow a certain amount of randomness in both systems over many runs. In the case of the Levine model, there is a certain probability of a pacemaker firing per timestep. In the Goldbeter model, the time offsets on the developmental path are randomly allocated. In both cases, pacemakers may randomly emerge in addition to the fixed ones. In the Goldbeter system, the fixed pacemakers are given a time advantage over the random pacemakers, while the small additional number in the Levine system makes the setup more comparable in this respect to the Goldbeter system. The resulting spiral occupancy images further highlight the dramatic differences between these models, with the clear Voronoi diagram-type tracing of the spiral tips in the Goldbeter model in contrast to the diffuse anticorrelation in the Levine model, which becomes clearer with clustered pacemaker elements, as in Fig 3A.

The different mathematical descriptions of excitable systems shape the spatial distribution of pattern elements in systematically different ways. More quantitative methods for exploring these constraints (via point process statistics; cf. [55]) have been discussed in [25]. Even the computation of correlation coefficients between pattern events can provide a first quantitative indication of their spatial relationships: in Fig 3D we compute such coefficients for the Goldbeter model with varying values of the Gaussian filter applied to the datasets. Using a fixed value of the quantity of the time offset on the developmental path, Δ, over 100 runs, we plot the mean values of the Spearman correlation coefficients against the value of the Gaussian filter (error bars are the standard error of the mean). Although the resulting quantities are small, they reflect the qualitative relationships observed in the event plots. We note a positive correlation between target wave origin occupancy and time offset, a negative relationship between spiral tip occupancy and target origin occupancy, and an apparent resulting anticorrelation between spiral tips and time offsets that is a maximum at our intermediate spatial scale and becomes less pronounced at larger scales.

How can these findings be put to use for a deeper understanding of the biological system? Experimental evidence for *Dictyostelium* suggests that many of these relationships, in particular, presence of a distinct target wave phase preceding the spiral wave patterns, are dependent on the biological strain [56]. Here, combining these sets of information, the geometric signatures of pattern events arising in a specific model and the dependence of these geometric constraints on experimental conditions, has the potential of revealing the intracellular mechanisms regulating the large-scale macroscopic, collective pattern states. This new avenue of research at the interface of computational systems biology and pattern formation is what we were referring to earlier as patterns serving as "microscopes" for the underlying regulatory principles.

We have seen that the distribution of cell properties affects the distribution of pattern elements and thus the details of the asymptotic pattern. How can variability, as a parameter measuring the amount of cell-to-cell differences, affect the patterns themselves? In [48] it was shown that the density of spiral waves changes with variability in a resonance-like fashion, resembling the classical phenomenon of diversity-induced stochastic resonance [57,58]. The underlying mechanisms here are a strong dependence of the competition between target waves and spiral waves on variability.

Additionally, the core idea of patterns as functionally important and evolutionarily advantageous collective states leads us to expect deep relationships between regulatory components and properties of spatiotemporal patterns. For the case of *Dictyostelium*, such relations should also reveal themselves in dedicated experiments, e.g., by varying the amount of cell-to-cell variability and studying the effect on pattern formation or by altering levels of specific regulatory components by mutagenesis and observing differences in pattern formation to the wild-type.

Furthermore, the findings summarized here can serve as a theoretical framework for reverse-engineering the fundamental regulatory mechanisms underlying the observed spatiotemporal patterns.

## Outlook

We have here introduced the principles of pattern predictability in biological systems, illustrated by a range of mathematical models and real experimental data. Taken together, the results described and reviewed here provide strong evidence supporting the general hypothesis that single-element properties are systematically mapped onto patterns and thus conserved across processes of self-organization (as opposed to being enslaved and "deleted" by the collective). The results reviewed here show that the initial properties of potentially very few cells have a driving influence on the resulting asymptotic collective state of the colony.

Although most of our examples so far have discussed pattern formation on regular lattices, the concept of pattern predictability can, in principle, be extended to other topological architectures, such as networks. The study by Marr and Hutt [59] is an example of how variability can reorganize patterns on networks: shortcuts inserted into a regular (ring-like) network architecture induce a transition from Wolfram class IV dynamics to Wolfram class III dynamics in binary cellular automata on graphs. The “variability” in these “small-world graphs” [60] deviating from regular networks lies in the degree (number of neighbors) of each element. With the work on Turing patterns on graphs [4] and related work [61,62], a new paradigm for the interpretation of dynamics on graphs is currently emerging: topology-compatible “collective modes” that establish themselves in a graph due to the interplay of topological and dynamical parameters. Waves organizing around hubs (highly connected nodes) are a striking example of such collective modes [63]. Networks are increasingly recognized as a powerful and intuitive way to represent real biological processes (see, e.g. [64,65]), with a vast range of applications extending from gene metabolism [66] to ecological processes [67].

We would like to acknowledge two areas not covered by our review: (i) the rich literature on cardiac dynamics, which is an important field of application for the study of spiral waves, is not discussed in detail. (ii) We have focused on spatially discrete models, as they provide a convenient framework for incorporating biological variability (by assigning a specific cell property to each spatial lattice site). Other work that very successfully uses partial differential equations for analyzing *Dictyostelium* pattern formation (e.g., [68]) is therefore not discussed in detail.

In the specific examples of spiral wave patterns discussed above, we want to highlight the importance of biological variability and the regulatory information contained in spatiotemporal patterns. However, we do not want to suggest that the complex relationship between the nonlinearities of the model, the nature and spatial distribution of cell properties, and the resulting spatiotemporal patterns is well understood and can lead to unique conclusions (e.g., about the relevant nonlinearities, given the distribution of cell properties). The details of this relationship remain far from clear.

Understanding the role of variability in pattern formation relies on disentangling the role of discrete element properties from that which may be an innate property of the system even without discretization, as is the case for homogeneous systems, such as the example explored in [50]. However, exploring the role of variability is appropriate in biological systems, as discreteness is an inevitable constituent of such systems: they are all composed of individual, interacting parts, whether at the molecular or the organismal scale.

Incorporating the spatiotemporal organization of biological systems is a major challenge for systems biology. The field is now making the transition from a purely temporal understanding of biological processes (the "well-stirred test-tube" perspective) to full spatiotemporal descriptions (see e.g., [69–71]). This, together with recent findings on the theoretical side, has reinvigorated the interest in classical models of spatiotemporal pattern formation.

One of the aims of systems biology is to establish the architectures and kinetics of signaling pathways and intracellular regulations in an iterative process between modelling and experiment. However, systems biology currently fails to exploit the very large pool of macroscopic observations represented by spatiotemporal patterns, which potentially provide important insight into intracellular regulation processes. Spatiotemporal patterns form within single cells or in a population of cells according to the intrinsic laws of protein–protein interactions, intracellular feedback loops and (on the multicellular level) cell–cell communication. The patterns change systematically with the parameters of regulation. When guided by a suitable mathematical model, the detailed layout of spatial and spatiotemporal patterns can reveal the properties of the underlying regulatory system.

The very recent publication by [72] is an ideal case study of this capacity of self-organized spatiotemporal patterns. By representing the intricate regulatory network responsible for the chemotactic response of individual *Dictyostelium* cells as a stylized excitable system (mathematically formulated as interconnected FitzHugh-Nagumo oscillators), a wealth of experimental information can be qualitatively explained, including the dynamics of adenylyl cyclase A in response to steps of external cAMP and the reason for the production of extracellular phosphodiesterases.

We are convinced that the general framework of pattern predictability has a vast range of additional applications, beyond the systems shown in Fig 1A. Analyzing pattern predictability can be envisioned, e.g., for cell sorting in in *Dictyostelium* [73], wound healing [74,75], prediction of Turing patterns [3], design of patterns in a broad range of self-organized processes and prediction of patterns in social and socioeconomical systems [76].

## Background

### Theoretical investigations of biological variability

Over several decades, a range of theoretical investigations has shown that variability can play an essential role in achieving a qualitative understanding of the processes at hand. The corresponding theoretical approaches range from variability as a driving force in evolutionary processes [77] to the generation of endogenous circadian oscillations from fast dynamics [78]. The vast field of research on synchronization in networks of nonlinear oscillators under the influence of variability and stochastic contributions has been described, e.g., from the point of view of synchronization [79] and from the network point of view [80].

The effect of variability on spatiotemporal chaos has been studied in [81–83]. Additionally, various numerical investigations have explored variability-induced pattern formation, compared to and in cooperation with noise using arrays of coupled oscillators [28,58,84]. The fact that, similarly to noise, variability can induce patterns or trigger a transition from one pattern to another has been a focus of research in the 1990s and early 2000s. In the more theoretically oriented studies, our biologically motivated term "variability" often appears as “diversity,” “disorder,” or “quenched noise.” For coupled FHN oscillators, it has been shown that variability can induce both a phase transition from oscillatory to excitable behavior [28] and, in a subexcitable system, pattern formation in the form of spiral waves of excitation [58]. Pattern complexity has been shown to be highest at intermediate variability [84]. Such studies culminated in the phenomenon of diversity-induced resonance [57,58], where the response of an excitable or bistable system to a subthreshold stimulus is optimal at intermediate levels of diversity. In [85] such a diversity-induced resonance was found in simulations of calcium dynamics, showing that calcium waves propagate optimally at intermediate cell-to-cell variability.

In recent work on excitable dynamics and spiral wave patterns, there is a strong trend to take into account realistic structural geometries in order to better understand the observed spatiotemporal patterns (see, e.g., [86]). On the methodological side, target and spiral wave identification techniques have been developed and implemented [49,87,88] and simulation methods have been advanced [89]. The authors of [90] also discuss how spiral tip identification in the FHN model influences the reconstructed patterns of meandering spiral waves. The use of generalized recurrence plots for reconstructing the phase diagram of nonlinear spatiotemporal systems from a limited set of observations has been described by [91]. In spite of its historical roots in the 1960s and 1970s, the explanation of spiral wave patterns still receives an enormous amount of scientific attention. The reason is 2-fold: (1) Our understanding of pattern formation processes still has severe gaps on the theoretical side; in particular, the routes from homogeneous patterns to fully established spiral waves are surprisingly dependent on the details of the system at hand. (2) It is currently being noticed that (precisely due to the dependence of patterns on the regulatory details within the system) a deep and detailed analysis of spiral wave patterns can help access the underlying principles of regulation. Detailed theoretical studies have provided evidence that the correlations between cell property distributions and patterns strongly depend on the regulatory mechanisms at the level of individual cells (e.g., comparing the results from [37] and [25]).

Returning to the purely theoretical description of properties of spatiotemporal patterns, it would of course be a major step forward if some aspects of the predictability of patterns from cell properties could also be understood analytically. Two approaches are particularly promising for tackling this question. Firstly, in [92] a mathematical framework has been developed for describing diffusion and annihilation of spiral wave tips within a simple kinematical model. Secondly, in [93] a response theory of (spiral wave) patterns under small perturbations has been formulated, in particular, the sensitivity of the spiral’s drift velocity.

Even in studies in which medium heterogeneity in the system is taken explicitly into account (e.g., [94]), the correlation between heterogeneity and the distribution properties of spiral waves is not discussed. We believe that this link could convey important insights into the prediction of spiral wave events, and on a wider level, the relationship between heterogeneity and system behavior has many additional practical applications in fields ranging from engineering to disease outbreak prevention. The strong practical interest in understanding spiral wave patterns in cardiac tissue has also led to a range of theoretical studies on the pinning of spiral waves by properties of the medium [95–98].

### *Dictyostelium* pattern formation

A model system for which regulation by variability could be a principal mechanism (see, e.g., [7,40]) is the slime mold *Dictyostelium discoideum*. In this paradigmatic example of biological pattern formation, individual amoeba cells aggregate under the influence of the chemotactic signal cAMP and form a multicellular organism [99]. This process is initiated by nutrient deprivation; this causes single cells to emit cAMP into their environment. These molecules are detected by neighboring cells via highly specific surface receptors [100], initiating the intracellular autocatalytic synthesis of additional cAMP by the enzyme adenylyl cyclase (ACA) and its subsequent secretion into the environment. Time-delayed receptor desensitization and halting of ACA activity are involved in the following refractory period. Extracellular cAMP is degraded by membrane-bound and secreted phosphodiesterase, which is, on the other hand, regulated by its inhibitor. The coupling of the underlying reaction kinetics with diffusion results in wave propagation. As long as the local cAMP concentration increases with time, the cells react with positive chemotaxis, resulting in periodic movement perpendicular to the wave front, i.e., towards the origin of the chemical signal.

As more details become available concerning the molecular network responsible for cAMP oscillations in *Dictyostelium* (e.g., [101,102]), modelling efforts increase in their corresponding complexity (see, e.g., [103,104], which are typical of this transition towards a more "systems biology" approach). In terms of the local *Dictyostelium* response to the cAMP gradient, progress is still being made in the understanding of the small-scale decisions underlying pattern formation [105]. Another evolving avenue of interest is *Dictyostelium*’s contribution to understanding the origins of multicellularity [106,107]; such efforts are spurred on by the sequencing of *Dictyostelium discoideum*’s genome [108]. These recent examples illustrate the continually increasing, vast amount of research that has been done over more than four decades into many aspects of the *Dictyostelium* life cycle. In particular, several findings of the last few years have added new ideas to the view that in the case of *Dictyostelium*, biological variability is responsible for certain stages of symmetry breaking in the usual course of the developmental cycle (local pattern initiation, spatial distribution of cell streams, and distribution and proportions of differentiated cell types).

In the work by the authors of [33], the strength of a regulatory feedback loop is related to the spatial density of spiral wave patterns in cell colonies of *Dictyostelium*. By studying mutants in key components of the regulatory feedback loop, the authors systematically varied this intrinsic parameter and observed how the spatiotemporal patterns changed accordingly. In order to display the systematics of their finding more clearly, the authors of [33] simulate patterns using a simple cellular-automaton based model, in which the feedback strength (i.e., the cAMP pulse-dependent increase in excitability) appears as an explicit parameter. Their observable is the spatial frequency of spiral waves, which they obtain by counting the phase singularities in their spatiotemporal patterns. Remarkably, the intermediate feedback strength found in wild-type cells turns out to produce an optimal (i.e., minimal) number of phase singularities compared to higher and lower feedback strength mutants, respectively. In the extreme case, in which feedback is constantly absent, no stable spiral wave pattern evolves. This observation has two implications. Firstly, aggregation territory size is optimized by the pulse-dependent development of feedback strength. For the wild type, this allows for optimally sized basins of attraction for the consecutive aggregation process of the cells leading to the multicellular organism capable of spore generation and thus completing the developmental cycle of *Dictyostelium*. Secondly, wave geometry is determined by feedback strength. Spiral waves seem to be favored by the system compared to target waves, although both wave geometries result in fruiting bodies for the experimental system. The biological advantage of spiral-based signaling is that spirals are self-sustaining continuous structures, which preserve their stability, to a certain extent outside the excitable regime. This allows for maintenance of the aggregation process under developmentally and environmentally conditioned changes. In contrast, target waves require the periodic activity of oscillatory regions (i.e. pacemakers).

Strong support for the hypothesis that cell properties can indeed affect the collective patterns in *Dictyostelium* has come from the recent observation that the direction and magnitude of a cell’s response to a signal pulse is an individual cell property, which remains constant in time [29]. In that work the behavior of single cells under periodic cAMP signals was analyzed and it is observed that the characteristics of the gradient sensing response of an individual cell at a certain time point strongly correlate with those of the same cell at a later time point. A recent paper [15] provides further support for our hypotheses, demonstrating that *Dictyostelium* cells’ internal cAMP concentrations oscillate at a frequency determined by intracellular machinery. The authors found that experimentally observed rhythmic cAMP synthesis could only be replicated successfully in their mathematical model when the stochastic pulsing of individual cells in response to subthreshold cAMP levels was included in the model. The implication is that organized group dynamics in a *Dictyostelium* population depend on the random behavior of individual cells (see also [109]). Another study finds that isogenic *Dictyostelium* cells have diverse sensitivities to cAMP, and that this may facilitate collective behavior [110]. Similarly, in [72] the roles of noise and variability in *Dictyostelium* cAMP signaling were explored in a phenomenological FitzHugh-Nagumo model. Here, variability was incorporated in the form of individual cells’ thresholds of response to extracellular cAMP. The results suggested that stochastic noise can account for population-level cAMP oscillations, while variability in this cell property alone cannot. These recent works, in addition to greatly enhancing the experimental accessibility of the system (via the direct measurement of cAMP concentrations), emphasize the role of individual cells and the debate whether it is the activity of dedicated pacemaker cells or the spontaneous firing of random cells which constitutes the main driving mechanisms behind the initiation of patterns during this early phase (see also [111]). While the work from [15] emphasizes the latter aspect, the results of [29] suggest an important role for the former, and it is likely that both noise and variability interact in the shaping of spatiotemporal patterns.

These diverse sets of questions, all of which are related to biological variability and their influence on pattern formation, illustrate the importance and timeliness of establishing a biology-oriented perspective on spatiotemporal patterns.

In the following, we will focus on two additional mathematical models. The first is a highly detailed model of pattern formation in *Dictyostelium*, which was initially formulated in [112]; we also refer to this model as the "Goldbeter" model. This model incorporates relevant biological details, such as the relay of suprathreshold cAMP pulses and autonomous cAMP oscillations, and the phosphorylation-dependent modification of the cAMP receptor, in the same way that current modeling attempts in systems biology would (see, e.g., [104,113]). Standard model reduction techniques (such as time scale separation) yielded a three-dimensional model that has also been elegantly used to analyze spatiotemporal patterns [40]. The dynamical variables are the fraction of active cAMP receptors and the concentrations of external and intracellular cAMP, respectively (see "Models" for details).

As in many such modeling situations, one now has the choice to either explore the biological details of these spatiotemporal patterns or, alternatively, aim at understanding the generic features. The second model to be discussed in detail in the following is therefore an array of coupled FitzHugh-Nagumo oscillators as a generic model for spatiotemporal patterns arising from excitable dynamics [53,54].

A novel ingredient organizing the interplay between biological variability and the resulting patterns in a system is the concept of a "developmental path." This is a specific parameter drift with time which couples small cell-to-cell property differences to the cell’s dynamical behavior, thus turning them into "organizers" of the spatiotemporal patterns. The "developmental path" concept was proposed by Lauzeral and coworkers [40], in the context of the model of *Dictyostelium discoideum* pattern formation developed by Martiel and Goldbeter [112] (see also [114]). It was created as a hypothetical mechanism of the development of heterogeneity from homogeneous conditions for the synthesis of spiral waves of cAMP in *Dictyostelium*. According to this concept, the cells in a population of *Dictyostelium* undergo time-dependent changes in cell properties, and intercellular variation in these properties places the cells at differing points along this developmental path. The path in [40] results from sigmoidal variations in the maximum activity of adenylate cyclase and the rate of extracellular cAMP degradation. The variation from cell to cell in the cell cycle when starvation begins is a possible origin of the different time offsets of the cells on the path. Such a path would be expected to occur in a higher-dimensional space in reality, but even in this simple form can provide an adequate representation of the actual biological dynamics. We consider the developmental path form of the Goldbeter model as an example of predicting patterns arising from a biologically plausible source of variability. Furthermore, we discuss the extension of the concept to the FHN model, showing that the principle is versatile and can be applied to diverse biological models as a method of generating heterogeneity.

### Models

Here we describe the models used throughout this review. The symbol ∇^2^ represents the discretized Laplacian for 2-D diffusion, and we use the five-point Laplacian except in the case of the Levine model, where the eight-point Laplacian is used. *D* is the value of the respective diffusion coefficient.

#### Schnakenberg model

This simple model [2] appears in Fig 1A, as an example of a driver of Turing-type patterns. Here, *c*_1_ = 0.05, *c*_-1_ = 1.0, *c*_3_ = 1.0, *D*_*u*_ = 1.0, *D*_*v*_ = 20.0, and *γ* = 2.0. The value of the parameter *c*_2_ determines whether the system gives rise to spots (*c*_2_ = 1.00) or stripes (*c*_2_ = 1.57). The initial values of *u* and *v* are randomly varied between -0.01 and 0.01.

$$\begin{array}{ll} \frac{\partial u}{\partial t} & \;=\;\gamma (c_{1}-c_{-1}u+c_{3}u^{2}v)+D_{u}\nabla^{2}u\\ \frac{\partial v}{\partial t} & \;=\;\gamma (c_{2}-c_{3}u^{2}v)+D_{v}\nabla^{2}v \end{array}$$ (1)

#### FitzHugh-Nagumo model

The FitzHugh-Nagumo (FHN) model [53,54] is widely used in studies of excitable media, as it is a low-dimensional model that can display a wide range of dynamics, including excitable, oscillatory, quiescent, and bistable behavior. Here it provides examples of target and spiral wave types (Fig 1A), spiral formation from a target wave due to interruption of the wavefront by less excitable elements (S1 Fig), and as an example of model-specific pattern events (S2 Fig). The FHN equations describing a 2-D lattice with diffusive coupling of the elements are:

$$\begin{array}{ll} \frac{\partial u_{i,j}}{\partial t} & \;=\;\frac{1}{\varepsilon }((a-u_{i,j})(u_{i,j}-1)u_{i,j}-v_{i,j})+D\nabla^{2}u_{i,j}\\ \frac{dv_{i,j}}{dt} & \;=\;bu_{i,j}-\gamma v_{i,j}+c_{i,j}(t) \end{array}$$ (2)

where *u*_*i*,*j*_ represents the voltage-like variable at each lattice site and *v*_*i*,*j*_ is the recovery variable. *c*_*i*,*j*_(*t*) is the parameter that may be subject to variability, either through a Gaussian distribution (“static” variant) or via a developmental path. The static variant appears in Fig 1A, S1 and S2A Figs, while the developmental-path variant appears in S2B Fig.

Each FHN element may be excitable, oscillatory, or in a non-excitable steady state, depending on the model parameters. Here, the parameters have the values *a* = -1, *g* = *b* = 0.12, *ε* = 1 and *D* = 0.1. A value of Δ*x* = 1.0 is used for the discretization.

For the static variant, there is no developmental path. For Fig 1A, *c* = 0.025 for the target wave and *c* = 0.021 for the spiral wave. For S1 Fig, *c* = 0.024 and an additional fraction equal to 0.400 of all elements are assigned *c* = 0.030, which is of lower excitability. In Fig 1A and S1 Fig, the central pacemaker element is assigned *c* = 0.000. For S2A Fig, values of *c* are randomly drawn from a Gaussian distribution, with mean value 0.024 and standard deviation 0.006. Varying the value of c changes the dynamical properties of the element; in S2A Fig the elements are mainly oscillatory, but some are also excitable and steady-state.

The developmental-path variant is analogous to the corresponding Goldbeter model, but incorporates variation in only one parameter, *c*:

$$c_{i,j}(t)\;=\;c_{0}\text{tanh}[\frac{t-t_{c}+\Delta t_{i,j}}{T_{c}}],$$ (3)

where the time offsets Δ*t*_*i*,*j*_ differ between elements (see Eq 4) and *t* is the “global time.” The value of *c*_0_ determines the dynamical regimes traversed in the temporal evolution of *c*_*i*,*j*_(*t*). Here it has the value 0.02, and the lattice values of *c*_*i*,*j*_(*t*) vary between approximately -0.02 at the start of the developmental paths and 0.02 at the end, with a value of *c*_*i*,*j*_(*t*)≈0 resulting in the maximal oscillatory frequency. The parameters *t*_*c*_ and *T*_*c*_ describe the shape of the path in *c*, here *t*_*c*_ = 1000 and *T*_c_ = 50.

Each FHN element is assigned a time offset Δ*t*_*i*,*j*_ on this path, which distributes the values of *c* over the lattice. The time offsets are created as above:

$$P(\Delta t_{i,j})\;=\;\frac{1}{\Delta }\text{exp}\left(-\frac{\Delta t_{i,j}}{\Delta }\right),$$ (4)

where Δ is a desynchronization parameter which controls the spread of time offsets Δ*t*_*i*,*j*_; here Δ = 500.

#### Three-state cellular automaton encoding excitable dynamics

This model appears in Fig 1C as an example of the development of a spiral wave from an open-ended planar wavefront. It consists of three discrete states for each node (susceptible *S*, excited *E*, refractory *R*), which are updated synchronously in discrete time steps according to a set of update rules allowing for signal propagation: (1) a susceptible node *S* becomes an excited node *E*, when a direct neighbor is in the excited state; (2) an excited node *E* enters the refractory state *R*; (3) a node regenerates (*R*→*S*) after *r* time steps. The parameter *r* is the deterministic refractory period of the system; here, *r = * 1.

In previous investigations [63,115] a stochastic version of the model has been explored, where spontaneous excitations could occur at random with a rate *f*, and the deterministic refractory period *r* was substituted by a stochastic recovery rate *p*. Such minimal models of an excitable system have a rich history in biological modeling. The stochastic version was first introduced in a simpler variant called the "forest fire model” [116] and subsequently expanded by Drossel and Schwabl [117] who also introduced the rate of spontaneous excitations, *f* (the "lightning probability” in their terminology). In this form it was originally applied to regular architectures in studies of self-organized criticality. Other variants of three-state excitable dynamics have been used to describe epidemic spreading (see, e.g., [118,119]).

#### Levine model from [7] and [33]

Here we discuss the "Levine" model of *Dictyostelium* pattern formation introduced in [7] (see also [33,37]). We will use the form from [33]. This model appears in Figs 1C, 2B, 3A and 3B. The model is a hybrid cellular automaton-ODE model for *Dictyostelium*, in which the cells have discrete internal states coupled to two continuous external variables: the local values of extracellular cAMP concentration and excitability. The rates of change of these variables are

$$\begin{array}{ll} \frac{\partial c_{i,j}}{\partial t} & \;=\;-\Gamma c_{i,j}+r_{F}s_{i,j}(t)+D\nabla^{2}c_{i,j}\\ \frac{dE_{i,j}}{dt} & \;=\;\eta +\beta c_{i,j} \end{array}$$ (5)

where *c* is the extracellular cAMP concentration, Γ is the constant of extracellular degradation of cAMP mediated by phosphodiesterase and *r*_*F*_ is the rate of cAMP production. Additionally, *s*_*i*,*j*_ is the internal cell state which controls cAMP production, taking the value of 1 for a firing state and 0 for a quiescent state, *η* is the intrinsic excitability increase and *β* is the genetic feedback factor which affects the sensitivity to signals from other cells.

The excitability increases monotonically and has an upper limit *e*_*max*_. In the ready and relative refractory states, activation can take place if the cAMP concentration exceeds the threshold *t*_*i*,*j*_:

$$t_{i,j}(\tau )\;=\;\left[c_{max}-A\frac{\tau }{\tau +T_{ARP}}\right]\left(1-E_{i,j}\right)$$ (6)

where *τ* is the time elapsed since the cell entered the relative refractory phase and has a maximum value of *T*_*RRP*_ for the ready state, and *A* = (*T*_*RRP*_+*T*_*ARP*_)·(*c*_*max*_-*c*_*min*_)/*T*_*RRP*_). Here, *T*_*RRP*_ and *T*_*ARP*_ are the relative and absolute refractory periods, respectively. A pacemaker has a certain probability *p*_*F*_ of firing per time step. The resulting random firing events lead to amplification of the heterogeneity of excitability in the system through feedback. For Fig 1C, pacemakers emerge randomly, as in [33] and the fraction of pacemaking elements *ε* = 0.50, *β* = 0.20, the starting excitability = *e*_*max*_/2 and *p*_*F*_ = 0.002. For Figs 2A, 3A and 3B, static distributions of pacemakers are used and the starting excitability = *η* = 0.0. For Figs 2A and 3A, *β* = 0.20, *p*_*F*_ = 0.002 and *ε* = 0.195 and 0.190 respectively. For Fig 3B, *β* = 0.30, *ε* = 0.001 (in addition to the five assigned pacemakers) and *p*_*F*_ = 0.05. Other parameter settings are as in [33].

#### Goldbeter model from [40,112]

This model of *Dictyostelium* pattern formation appears in Figs 2B and 3C, and in the accompanying event plot animation (S1 Video). It was developed by Martiel and Goldbeter [112] (see also [114]). We here discuss the reduced, three-dimensional version of the original model with nine dynamic variables, both from [112]. Each lattice point represents a group of ten cells whose properties are synchronized.

The three dynamic variables are the total fraction of active cAMP receptor (*ρ*_*T*_) and the normalized concentrations of intracellular (*β*) and extracellular (*γ*) cAMP:

$$\begin{array}{ll} \frac{d\rho_{T}}{dt} & \;=\;-f_{1}(\gamma )\rho_{T}+f_{2}(\gamma )(1-\rho_{T}),\\ \frac{d\beta }{dt} & \;=\;q\sigma \Phi (\rho_{T},\gamma ,\alpha )-(k_{i}+k_{t})\beta ,\\ \frac{\partial \gamma }{\partial t} & \;=\;(k_{t}\beta /h)-k_{e}\gamma +D_{\gamma }\nabla^{2}\gamma , \end{array}$$ (7)

with

$$\begin{array}{ll} f_{1}(\gamma ) & \;=\;\frac{k_{1}+k_{2}\gamma }{1+\gamma },f_{2}(\gamma )\;=\;\frac{k_{1}L_{1}+k_{2}L_{2}c\gamma }{1+c\gamma }\\ \Phi (\rho_{T},\gamma ,\alpha ) & \;=\;\frac{\alpha (\lambda \theta +\varepsilon Y^{2})}{1+\alpha +\varepsilon Y^{2}(1+\alpha )},Y\;=\;\frac{\rho_{T}\gamma }{1+\gamma }. \end{array}$$ (8)

This model is based on reversible desensitization of the cAMP receptors on the surface of cells. Extracellular cAMP may bind to the active receptor; this activates adenylate cyclase *σ*, converting intracellular ATP to cAMP. The activity of the receptors depends upon the extracellular cAMP concentration and the fraction of active receptors. Intracellular cAMP diffuses out of the cell and can be hydrolyzed by intra- or extracellular phosphodiesterase *k*_*e*_.

The "developmental path" concept was proposed by Lauzeral and coworkers [40], in the context of the three-dimensional model. According to this mechanism, specified cell properties follow a defined trajectory over time, with this variation leading cells successively through quiescent, excitable, oscillatory and excitable regimes of dynamical behavior. Desynchronization of the cells’ properties on this path then provides the necessary cell-to-cell differences for spiral waves to form. This path incorporates variation in adenylate cyclase and phosphodiesterase, in correspondence to experimental observations. The developmental path concept thus provides a mechanism for the generation of variability from homogeneous initial conditions. This model was shown to successfully reproduce the sequence of macroscale patterning observed in experimental *Dictyostelium* colonies.

Here we discuss developmental path 3 of [40], in which *σ* and *k*_*e*_ are varied sigmoidally:

$$\begin{array}{ll} \sigma (t) & \;=\;\sigma_{av}+\sigma_{amp}\text{tanh}\left[\frac{t-t_{\sigma }+\Delta t_{i,j}}{t_{\sigma }}\right]\\ k_{e}(t) & \;=\;k_{av}+k_{amp}\text{tanh}\left[\frac{t-t_{k}+\Delta t_{i,j}}{T_{k}}\right]. \end{array}$$ (9)

where *t* is the "global time" and the parameters *t*_*σ*_ and *T*_*σ*_; and *t*_*k*_ and *T*_*k*_ describe the shape of the variations in *σ* and *k* respectively. The lattice elements (representing synchronized groups of cells) are assigned time offsets Δ*t*_*i*,*j*_ on this path, which distributes the values of *σ*(*t*) and *k*_e_(*t*) over the lattice. As in [40], each time offset is randomly drawn from an exponential distribution:

$$P(t_{i,j})\;=\;\frac{1}{\Delta }\text{exp}\left(-\frac{t_{i,j}}{\Delta }\right).$$ (10)

In Figs 2B, 3D, and the accompanying animation (S1 Video), Δ = 25; in 3C, Δ = 15. Other parameter settings are as described in [40].

## Supporting Information

S1 Fig Development of spiral waves from a central pacemaker in the FitzHugh-Nagumo (FHN) model due to the presence of elements of lower excitability, leading to curvature of the planar wavefront. The entirety of the lattice is excitable, but a fraction of 0.4 of the elements have lower excitability.
(EPS)

S2 Fig Pattern event sequences in "static" and "developmental-path" variants of the FHN model. Left to right: lattice snapshots with time and overlaid pattern events (red asterisks are target origins, blue and green diamonds are left- and right-handed spiral tips). A: Development of spiral waves from the interaction of target waves with areas of lower excitability in the "static" FHN model. B: Development of spiral waves from target waves in the FHN model with a developmental path. Spirals originate within the zones of target wave entrainment, apparently through the wave-in-wave mechanism.
(EPS)

S1 Video Animation of the spatiotemporal evolution of a lattice of "Goldbeter" elements, as their time offsets move along a developmental path in the parameters *σ* and *k*_*e*_. Pattern events are represented by red asterisks (target wave origins) and left- and right-handed spiral tips (blue and green diamonds, respectively). Left side: development of the *u*-field with overlaid detected events; right side: space-time plot of the pattern events.
(AVI)
